# Preservation of Vaccine Crusts

**Published:** 1871-04

**Authors:** David Stewart


### PRESERVATION OF VACCINE CRUSTS.

BY DAVID STEWART, M. D.

Vaccine lymph may be preserved during all the summer months, in any climate, by the following expedient, which I devised several years since: Immerse them in mercury, and keep the package in a cool cellar, or ice-house or well. No moisture can reach them, although the package is placed beneath the surface of water in the well, or sunk to the bottom thereof. Moreover, they will be dried more thoroughly when deeply imbedded in water, for manifest reasons, and not only protected from insects, but the peculiar animal, which forms at their expense, (invariably when they are otherwise stored away) and equals them in size ultimately, will not occur. In 1868, I preserved them thus successfully, in my cellar, from spring until autumn, by attaching a slice of cork to a thread, which facilitates its removal from a tube vial or "test tube," when forced down and confined by its own elasticity to the lower extremity; this slice of cork I marked with the date, &c., and then dropped upon it some melted beeswax, one drop of which is sufficient to attach the crust to one side of the disc of cork which suspends it, clear of the glass at the bottom, under a stratum of mercury which may be subsequently introduced until the tube is filled; but one inch of mercury I prefer, although much less may answer, though provided the cork is covered therewith; especially if (by the mouth) the pressure of the atmosphere is partly removed (sucked out) from its surface momentarily, as this is *more* than equivalent to the effect that would otherwise result if even twenty (20) inches of mercury were imposed. In other words, the vaccine is inclosed in a *quasi* Torricillian vacuum; and, moreover, any air on its surface is expanded and escapes above the stratum of mercury. Upon this principle, delicate anatomical preparations may be kept during the summer months in their original perfection, provided eremecausis has not commenced.—*Amer. Jour. of Phar.*

*Port Penn, Delaware, June 9th,* 1870.

